
JOURNAL INFORMATION
==============================
NLM Title Abbreviation: Wirtschaftsdienst
Journal ID: Wirtschaftsdienst
NLM Journal ID: 101086612

Wirtschaftsdienst (Hamburg, Germany : 1949)

ISSN: 0043-6275

ARTICLE INFORMATION
==============================
PMCID: PMC7813615
PMID: 33487764
DOI: 10.1007/s10273-021-2811-y
Article ID: 2811
Article version: 1
Subjects: Kurz Kommentiert

Wie geeignet ist Free to Choose?

COVID-19-Impfstrategie

COVID-19 vaccination strategy: How suitable is Free to Choose?

Beretta Edoardo edoardo.beretta@usi.ch

grid.29078.34 0000 0001 2203 2861 Wirtschaftswissenschaftliche Fakultät, Università della Svizzera italiana, Via Buffi 13, 6900 Lugano, Switzerland
Electronic publication date: 2021 Jan 19
Print publication date: 2021
Volume: 101
Issue: 1
First page: 5
Last page: 5
Copyright: © Der/die Autor(en) 2021
License: Open Access: Dieser Artikel wird unter der Creative Commons Namensnennung 4.0 International Lizenz (https://creativecommons.org/licenses/by/4.0/deed.de) veröffentlicht.
Open Access wird durch die ZBW — Leibniz-Informationszentrum Wirtschaft gefördert.
License URL: https://creativecommons.org/licenses/by/4.0/

issue-copyright-statement: © The Author(s) 2021

==============================
COVID-19-Impfstrategie

### Wie geeignet ist Free to Choose?

Als Milton und Rose Friedman 1980 ihr neoliberales Bekenntnis in „Free to choose. A personal statement" zusammenfassten, plädierten sie für das individuelle Recht, sich frei von (übertriebener) staatlicher Regulierung zu entfalten. 41 Jahre später inmitten einer der Pandemien mit vielen Todesopfern, die es im Verlauf des 20. Jahrhunderts gegeben hat (1 Mio. nach der Hongkong-Grippe (1968), 1,1 Mio. nach der Asiatischen Grippe (1957-1958) und bis zu 50 Mio. nach der Spanischen Grippe (1918-19)), verlautet es aus Regierungskreisen weltweit unisono, eine Impfung gegen SARS-CoV-2 solle nur auf freiwilliger Basis erfolgen. Etwas zugespitzt: Die Rückkehr zur „echten" Normalität und inwieweit sich das im Jahr 2020 laut World Economic Outlook des IWF um -4,4 % zurückgehende globale BIP erholen wird, hängt von der Impfbereitschaft der Bevölkerung ab. Trotz fehlenden Impfzwangs solle aber zum gemeinsamen Wohlergehen nach erreichter Herdenimmunität (d. h. 60 % bis 70 % der Bevölkerung geimpft) noch Überzeugungsarbeit durch Wissenschaft, Politik sowie Medien geleistet werden.

Derart gesprächs- oder verständnisbereit haben sich weltweite Entscheidungsträger:innen ceteris paribus (sprich: bei vergleichbarer Notwendigkeit des Schutzes der Bevölkerung) nicht gezeigt, als Lockdowns verordnet wurden mit Zwangsschließungen, Ausgangssperren, Quarantänepflichten oder, wie im italienischen Fall, Reiseverbote in andere Inlandsregionen und schriftliche Eigenerklärungen zur Erledigung zugelassener Geschäfte außerhalb der eigenen Wohnadresse. Obwohl bei der Impffrage nun das Free-to-choose-Prinzip gelten soll, ist es bei den bisherigen Maßnahmen durch Zwangsbeschlüsse ersetzt worden.

Trotz Freiwilligkeit wird die Bevölkerung zum „Don't call us, we call you" aufgefordert, zumal die Impfzentren sich nach Risikokategorien vorzuarbeiten haben. Dass eine höhere Risikostufe nicht mit einer größeren Impfbereitschaft einhergehen muss (und potenzielle Impfkandidat:innen aus anderen Risikogruppen länger zu warten haben), scheint hinnehmbar zu sein. Ebenso lässt sich trotz Freiwilligkeit der Impfstoff unter den verfügbaren nicht frei wählen, obwohl je nach Impftypus und/oder -dosierung ein Impfeffektivitätsgefälle von mehreren 10 Prozentpunkten herrscht. Und was würde geschehen, wenn ein einmalig Geimpfter den zweiten Impftermin nicht antreten sollte? Oder wie würde der Fall eines Impfkandidaten gehandhabt, der sich zunächst gegen eine Impfung entschlossen, sich dann umentschieden hat, seine Impfkategorie aber bereits nicht mehr an der Reihe sein sollte?

Es handelt sich hierbei um strategische Managementfragen, die über den Effizienzgrad bei der endgültigen Pandemiebekämpfung entscheiden. Und darüber, ob Free-to-choose-Prinzipien oder staatlich gelenkte Top-down-Entscheidungsansätze gelten sollen. Welche Herangehensweise man auch vorziehen sollte: Kohärenz bleibt auch bei knappen Impfdosen unerlässlich.

Anzusprechen ist auch, ob das jetzige „hybride" Free-to-choose-Prinzip eine Kostenbeteiligung von Impfverweigernden im Falle eigener Ansteckung (und/oder anderer) vorsehen sollte. Aus makroökonomischer Sicht wäre es schwer befürwortbar, dass die globale Wirtschaft – trotz milliardenhoher staatlicher Käufe von Impfdosen – weiterhin dem Ausfallrisiko nichtgeimpfter Arbeitskraft und den einhergehenden Quarantäne- und Gesundheitskosten ausgesetzt sein sollte. Aus mikroökonomischer Perspektive wären die Voraussetzungen für Free Riding gegeben, wenn Nichtimpfbereite nicht die Kosten ihrer Ansteckung (und/oder anderer) zu tragen hätten. Ein solcher finanzieller Ansatz wäre ein effektiveres und weniger polarisierendes Nudging als der jüngst vorgeschlagene Verzicht auf ein Intensivbett im Falle der Erkrankung. Auch die zuletzt diskutierte Möglichkeit, einen Impfpass vorzusehen, um Großveranstaltungen, Reisen usw. anzutreten, mag mit dem Free-to-choose-Prinzip vereinbar sein, ist aber nicht optimal, zumal die wirtschaftlichen Negativeffekte des potenziellen Abhandenkommens nichtgeimpfter Kundschaft auf andere (d. h. Unternehmen und Realwirtschaft) abgewälzt würden. Laut einer im November 2020 veröffentlichten Umfrage des Weltwirtschaftsforums haben sich 69 % der Befragten in Deutschland für eine baldige Impfung gegen COVID-19 ausgesprochen. In Frankreich waren es hingegen nur 54 %: Was ein derartiger Anteil für impfpassabhängige Konsumausgaben auch nur zeitweise bedeuten könnte, lässt sich erahnen.

Wenn die Bevölkerung „frei" entscheiden soll, darf es keinerlei Mechanismen geben, die einerseits eine dirigistische und zeitaufwändige Impfstoffverteilung vorsehen, andererseits unverantwortliche Entscheidungen nicht verfolgen. Es wird kommen, wie es kommen muss: Wenn der Nachahmeffekt nicht für eine Mindestimpfquote ausreicht, wird die Impfpflicht kommen. Dass dann schon wieder kostbare Zeit verstrichen sein wird, ist allerdings klar.

Edoardo Beretta
Università della Svizzera italiana
edoardo.beretta@usi.ch

© Der/die Autor:in(nen) 2021. Open Access: Dieser Artikel wird unter der Creative Commons Namensnennung 4.0 International Lizenz (https://creativecommons.org/licenses/by/4.0/deed.de) veröffentlicht.

Open Access wird durch die ZBW – Leibniz-Informationszentrum Wirtschaft gefördert.
